# Circular RNA METTL9 contributes to neuroinflammation following traumatic brain injury by complexing with astrocytic SND1

**DOI:** 10.1186/s12974-023-02716-x

**Published:** 2023-02-17

**Authors:** Chunling Huang, Lulu Sun, Chenyang Xiao, Wenjun You, Li Sun, Siye Wang, Zhijun Zhang, Su Liu

**Affiliations:** 1 Department of Rehabilitation Medicine, Affiliated Hospital of Nantong University, Medical School of Nantong University, Nantong, 226001 Jiangsu Province China; 2 Department of Human Anatomy, Medical School of Nantong University, Nantong, 226001 Jiangsu Province China; 3 Department of Geriatrics, Affiliated Nantong Rehabilitation Hospital of Nantong University, Nantong, 226001 Jiangsu Province China

**Keywords:** Circular RNA METTL9, Neuroinflammation, Traumatic brain injury, Astrocyte, Chemokines, SND1

## Abstract

**Background:**

Circular RNAs (circRNAs) are highly enriched in the central nervous system and have been implicated in neurodegenerative diseases. However, whether and how circRNAs contribute to the pathological processes induced by traumatic brain injury (TBI) has not been fully elucidated.

**Methods:**

We conducted a high-throughput RNA sequencing screen for well-conserved, differentially expressed circRNAs in the cortex of rats subjected to experimental TBI. Circular RNA METTL9 (circMETTL9) was ultimately identified as upregulated post-TBI and further characterized by RT-PCR and agarose gel electrophoresis, Sanger sequencing, and RNase R treatment. To examine potential involvement of circMETTL9 in neurodegeneration and loss of function following TBI, circMETTL9 expression in cortex was knocked-down by microinjection of a shcircMETTL9 adeno-associated virus. Neurological functions were evaluated in control, TBI, and TBI-KD rats using a modified neurological severity score, cognitive function using the Morris water maze test, and nerve cell apoptosis rate by TUNEL staining. Pull-down assays and mass spectrometry were conducted to identify circMETTL9-binding proteins. Co-localization of circMETTL9 and SND1 in astrocytes was examined by fluorescence in situ hybridization and immunofluorescence double staining. Changes in the expression levels of chemokines and SND1 were estimated by quantitative PCR and western blotting.

**Results:**

CircMETTL9 was significantly upregulated and peaked at 7 d in the cerebral cortex of TBI model rats, and it was abundantly expressed in astrocytes. We found that circMETTL9 knockdown significantly attenuated neurological dysfunction, cognitive impairment, and nerve cell apoptosis induced by TBI. CircMETTL9 directly bound to and increased the expression of SND1 in astrocytes, leading to the upregulation of CCL2, CXCL1, CCL3, CXCL3, and CXCL10, and ultimately to enhanced neuroinflammation.

**Conclusion:**

Altogether, we are the first to propose that circMETTL9 is a master regulator of neuroinflammation following TBI, and thus a major contributor to neurodegeneration and neurological dysfunction.

**Graphical Abstract:**

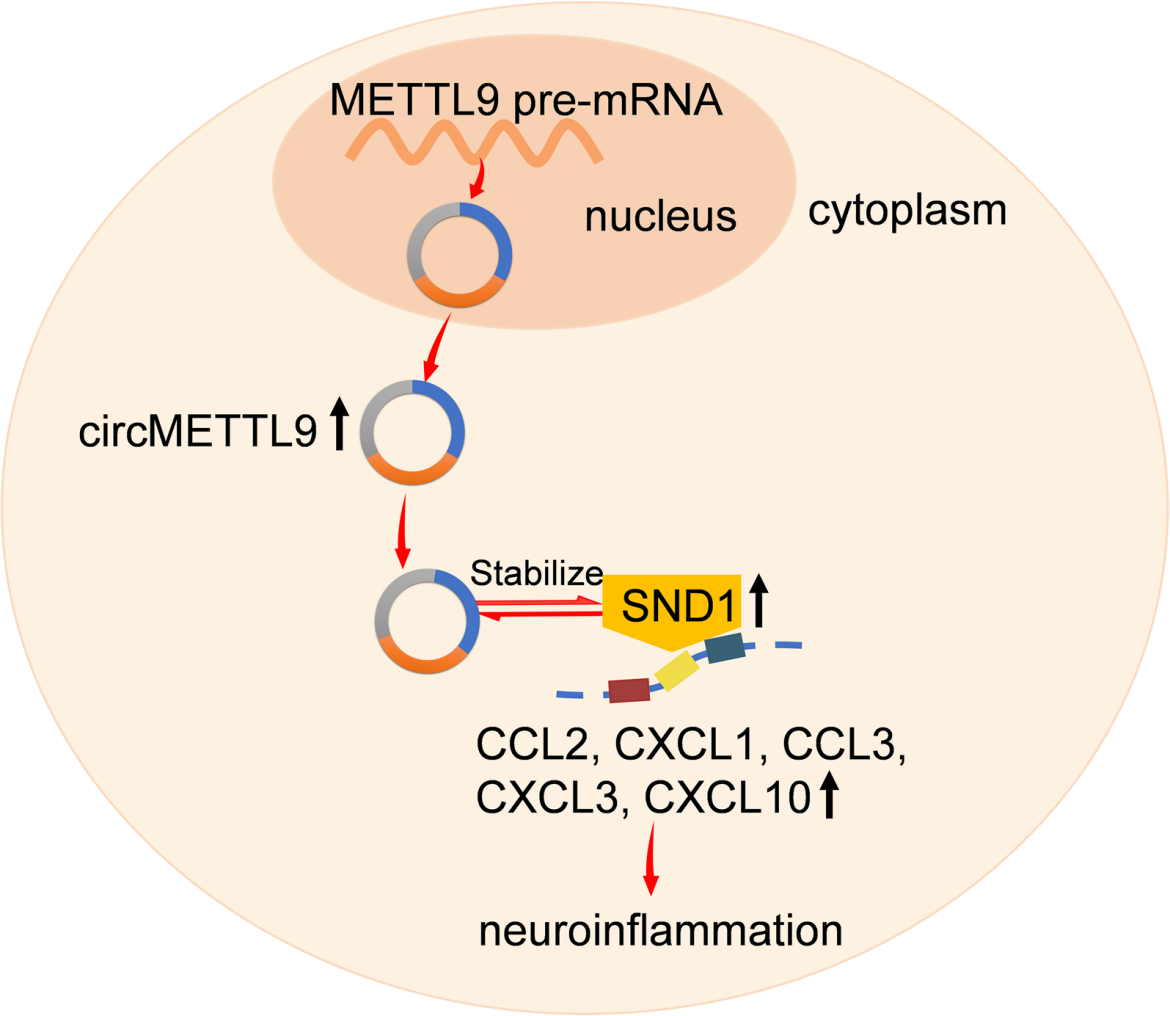

**Supplementary Information:**

The online version contains supplementary material available at 10.1186/s12974-023-02716-x.

## Introduction

Traumatic brain injuries (TBIs) resulting from traffic, industrial, and recreational accidents, falls, and military conflicts among other events are a major and growing cause of disability and death worldwide [1]. Survivors are often left with cognitive, motor, psychological, and emotional impairments that seriously reduce quality of life and place heavy burdens on families and healthcare systems [2, 3]. Typical pathophysiological characteristics of TBI include synaptic dysfunction, neuronal cell death, axonal damage, glial cell activation, neuroinflammation, and immune cell infiltration [4, 5]. Collectively, these processes extend both the region of neural damage and the spectrum of impairments beyond those resulting from the initial event, termed secondary injury. Therefore, there is an urgent need to identify the molecular mechanisms that exacerbate TBI and develop more effective treatments.

Circular RNAs (circRNAs) are a unique class of endogenous non-coding RNAs with a closed circular structure that are derived from exon skipping or back-splicing of precursor mRNA (pre-mRNA) [6]. These species are not susceptible to RNA exonuclease degradation, and thus are stably expressed, with particularly high levels of expression in the central nervous system [7]. Current research has revealed diverse functional roles for circRNAs, including regulation of source gene transcription [8], sponging of miRNAs, RNA-binding proteins (RBPs), and other circRNAs, and potential translation into peptides [9–12]. The spatiotemporal pattern of circRNA expression appears relatively constant in healthy brain, but there is accumulating evidence for altered expression in several disorders, including acute and chronic neurodegenerative disorders, that influences critical pathophysiological processes such as apoptosis, autophagy, angiogenesis, neuronal plasticity, and inflammation [13]. Thus, circRNAs may be novel treatment targets for central nervous system diseases. The aforementioned processes are strongly implicated in secondary injury following TBI, but the potential contributions of dysregulated circRNAs have not been investigated in great detail.

Neuroinflammation is essential for maintaining tissue homeostasis, but is also a major contributor to secondary injury post-TBI [14]. Therefore, it is vital to identify the precise molecular mechanisms regulating the neuroinflammatory response to TBI for treatment development. Several recent studies have implicated circRNAs in the regulation of neuroinflammation following TBI. For instance, the upregulation of circRNA chr8_87,859,283-87,904,548 in mouse cerebral cortex after experimental TBI was found to increase the expression of CXCR2 by sponging the species-specific microRNA (miRNA) mmu-let-7a-5p, resulting in enhanced neuroinflammation and disruption of neuronal functional recovery [15]. Alternatively, knockdown of circLrp1b in TBI rats was reported to suppress the production of pro-inflammatory cytokines interleukin (IL)-1β, IL-6, and tumor necrosis factor (TNF)-α, suggesting that different circRNAs have reciprocal effects on neuroinflammation [16]. However, the possible functional and molecular mechanisms by which circRNAs regulate TBI-induced inflammation are still unclear.

To address this question, we conducted high-throughput sequencing for differentially expressed circRNAs in the cortex of TBI model rats, and identified circMETTL9 (originating from the gene encoding methyltransferase like 9, or METTL9) as significantly upregulated. Furthermore, we found that knockdown of circMETTL9 in the cerebral cortex improved functional recovery following TBI, at least in part by suppressing pro-inflammatory chemokines signaling. We hypothesized that knockdown of circMETTL9 would reduce the expression of chemokines, thereby ameliorating neuroinflammation, resulting in improved neurobehavioral outcomes after TBI.

## Methods and materials

### Animals and experimental TBI

Male Sprague-Dawley (SD) rats weighing 230 to 270 g (about 8 weeks) were obtained from the Experimental Animal Center of Nantong University (Nantong, China) and housed in an experimental holding room with controlled temperature (23 ± 2 °C), humidity (40%), and 12 h/12 h light/dark cycle. Animals had ad libitum access to standard rodent chow and fresh water. All experimental procedures were approved by the Ethics Committee of Laboratory Animals of Nantong University (Approval No. IACUC20220310-1001) and were conducted according to the guidelines of the National Institutes of Health (NIH).

Traumatic brain injury was induced by controlled cortical impact (CCI) as described in our previous study [17]. Briefly, male SD rats were anesthetized under 4% isoflurane and placed into a stereotactic frame. A circular hole 6 mm in diameter was created in the cranium over the right parietal lobe (3.0 mm posterior to the bregma and 3.0 mm to the right of the midline) using a dental drill to expose the dura mater. Controlled cortical impact was performed using a 5-mm diameter impingement head at an impact speed of 4 m/s, deformation depth of 3.0 mm, and duration of 150 ms to cause severe TBI models. The cranial window and skin wound were then close and disinfected. Rats were placed next to a heater for recovery and then returned to the home cage. A second group of sham control rats were subjected to the same surgical treatment but without CCI.

### CircRNA sequencing

Total RNA was isolated from the cerebral cortex of sham and TBI model rats (*n* = 4 per group) using RNAiso Plus (TaKaRa, Tokyo, Japan) and evaluated for integrity using an Agilent 2100 Bioanalyzer automated electrophoresis system (Applied Biosystems, Carlsbad, CA, USA). Linear RNAs were digested using exonuclease (Epicentre Technologies, Madison, WI, USA) and circRNA sequencing was then performed using the Novaseq 6000 PE150 platform (Illumina). CircRNAs were mapped using STAR Chimeric Post (STARChip) alignment software [17] and then identified and quantified using the DCC program [18]. Differentially expressed genes were identified using edgeR.

### Adeno-associated virus injection

An adeno-associated virus (AAV) vector for circMETTL9 knockdown, AAV2/9-circMETTL9-T2A-GFP, was constructed and synthesized by HANBIO (2.7 × 10^12^ viral genomes/µL, Shanghai, China). A total of 100 rats were randomly divided into 4 groups: Sham group (*n* = 20), TBI group (*n* = 20), AAV2/9-shNC + TBI group (*n* = 30), and AAV2/9-shcircMETTL9 + TBI group (*n* = 30). AAV2/9-shNC is the control virus and AAV2/9-shcircMETTL9 is the virus which can knockdown of circMETTL9. Briefly, rats were anesthetized under 4% isoflurane and placed into a stereotactic frame. As in our previous study [17], three holes were created in the right parietal bone with a dental drill (1.5 mm posterior to bregma and 1.5 mm lateral to the midline; 1.5 mm posterior to bregma and 3 mm lateral to the midline; 3 mm posterior to bregma and 1.5 mm lateral to the midline) for injection of vehicle or virus (2 μL/hole) at 0.2 μL/min and a depth of 1.3 mm from the skull surface using a Hamilton syringe. After injection, the syringe needle was left in place for 10 min to ensure distribution of the virus.

### Behavioral assessment

The modified neurological severity score (mNSS) test battery assessing motor (muscle status, abnormal movement), sensory (visual, tactile and proprioceptive), reflex, and balance functions [18] was conducted on days 1, 3, and 7 following TBI or sham treatment. One point was scored for the inability to perform each test or for the lack of a tested reflex. A higher score (18 maximum) was indicative of more severe neurological dysfunction.

One day after CCI or sham treatment, rats were tested for hippocampus-dependent reference memory in the Morris water maze (MWM) test. The testing protocol consisted of a 4-day learning phase and subsequent spatial memory assessment on day 5. Each rat received 4 training trials per day (16 in total). Briefly, an individual rat was released from a random location facing the MWM wall and allowed to swim until it found the submerged escape platform. If a rat failed to find the platform within 90 s, it was guided their and allowed to stay for 20 s. The latency to find the platform was measured as an index of spatial learning. In the probe test of spatial memory on day 5, the platform was removed, and rats were released from the quadrant opposite to that of the former platform location. The number of crossings over the former platform location within 90 s was measured as an index of spatial memory.

### TUNEL staining

Brains containing the entire cortex were cut into 20-μm sections using a cryostat and apoptotic cells stained using the TUNEL BrightRed Apoptosis Detection Kit (A113-01, Vazyme, Nanjing, China) according to the manufacturer’s protocol. Sections were then treated with anti-fluorescence quenching tablets containing DAPI for counterstaining of nuclei. Staining was then imaged under fluorescence microscopy and quantified using ImageJ.

### Cell culture

Primary rat astrocytes were obtained from the cerebral cortices of neonatal rats as described [17]. Briefly, cortices were quickly isolated and placed in cold D-Hank’s buffer, carefully cleared of membranes and large blood vessels, then triturated into a cell suspension by passing through a 70 mesh filter. Cells were plated in high glucose medium containing 10% fetal bovine serum and cultured in a 5% CO_2_ incubator at 37 °C.

### Overexpression plasmid and small interfering (si)RNA transfection

Plasmids for overexpression of circMETTL9 and siRNAs for knockdown of circMETTL9 and SND1 in cultured astrocytes were obtained from GenePharma (Suzhou, China). Sequences are listed in Additional file 1: Table S1 and Additional file 2: Table S2.

### RNA pull-down and mass spectrometry

Biotin-labeled probes targeting the junction site of circMETTL9 were designed and synthesized by Obio Technology (Shanghai, China). Astrocytes were activated by treatment with lipopolysaccharide (LPS) for 12 h and lysed by probe sonication. Lysates were incubated with the probe to form RNA–protein complexes, which were then isolated by incubation with streptavidin-coated magnetic beads. Finally, complexes were eluted off the beads and RNA-binding proteins identified by silver staining and mass spectrometry. The probe sequences are listed in Additional file 3: Table S3.

### Fluorescence in situ hybridization (FISH) and immunostaining

FISH was performed in primary astrocytes and frozen sections of the cerebral cortex using a commercial kit from GenePharma (Shanghai, China) according to the manufacturer’s protocol. Astrocytes treated as indicated were fixed, permeabilized, and treated sequentially with 1 × streptavidin in 5% BSA at 37 °C for 30 min to block nonspecific binding and then with 2 × SSC at 37 °C for 30 min. A biotin-probe was denatured in a 75 °C water bath for 10 min, incubated with SA-Cy3 and hybridization buffer, and then added to cells under darkness at 37 °C for 16 h. Stained cells were washed several times with 2 × SSC, treated with anti-fluorescence quenching tablets containing DAPI (Solarbio, S2110) for nuclear counterstaining, and imaged under a confocal fluorescent microscope (Olympus, Tokyo, Japan). Frozen sections were first incubated with preheated denaturation solution at 78 °C for 8 min, and then FISH was conducted using the same steps as above. The rat circMETTL9 probe sequence is listed in Additional file 4: Table S4.

Combined FISH/immunostaining was performed as described previously [19]. Briefly, brain sections or cells were washed several times with 2 × SSC, blocked by incubation in goat serum at 37 °C for 1 h, and then immunolabeled by incubation with GFAP antibody (1:100; Proteintech, Rosemont, IL, USA; 16825-1-AP) or SND1 antibody (1:100; Proteintech, 60265-1-Ig) overnight at 4 °C. Samples were then incubated with corresponding secondary antibodies for 1 h, treated with anti-fluorescence quenching tablets containing DAPI for counterstaining, and imaged under a confocal fluorescence microscope.

### RNA isolation and quantitative polymerase chain reaction

Total RNA was isolated from cells and brain tissues using TRIzol Reagent (Vazyme Biotech, Nanjing, China) and reverse transcribed (1 μg RNA per reaction) into complementary DNA using the HiScript II Q Select RT SuperMix for qPCR (+ gDNA wiper) (R233-01, Vazyme, Nanjing, China). Quantitative (q)PCR was performed using the ChamQ SYBR qPCR Master Mix (High ROX Premixed) (Q341-02, Vazyme) and the primers listed in Additional file 5: Table S5, on a StepOnePlus™ real-time PCR system (Invitrogen, Carlsbad, CA, USA). Expression levels were calculated using the 2^−ΔΔCT^ method and normalized to β-actin expression.

### Western blotting

Lysate proteins were separated by polyacrylamide gel electrophoresis and transferred to PVDF membranes. Membranes were blocked with 5% non-fat skim milk for 3 h, incubated sequentially in primary antibody against SND1 (1:5000; Proteintech, 60265-1-Ig) or β-actin (1:5,000; Proteintech, 66009-1-Ig) at 4 °C overnight and then in secondary antibodies for 1 h, and treated with enhanced chemiluminescence reagent (Proteintech, PK10001). Protein band intensities were quantified using ImageJ.

### Statistical analysis

All results are expressed as mean ± standard error of the mean (SEM). Before analysis, the Shapiro–Wilk test was used to test the normality of the variables. Two group means were compared by Student’s* t*-test and more than two group means by one-way or two-way analysis of variance (ANOVA) followed by post hoc pair-wise comparisons using Tukey’s test (for one-way ANOVA) or Bonferroni's correction (for two-way ANOVA). *P* < 0.05 was considered statistically significant for all tests. All statistical analyses were performed using GraphPad Prism 9.1.0 (San Diego, CA, USA).

## Results

### Expression profiling of circRNAs in rat cerebral cortex after TBI

**Fig. 1:**
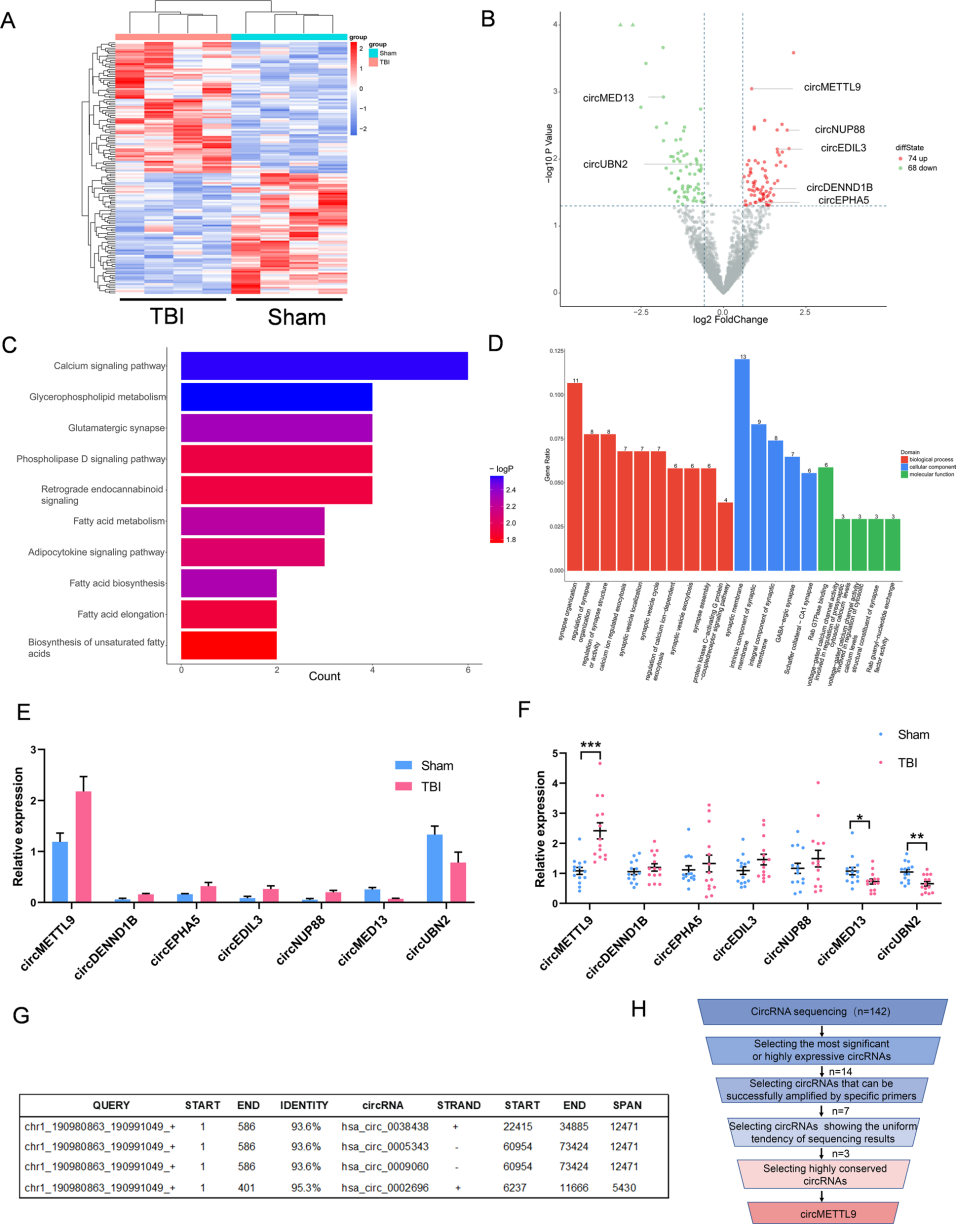
Upregulation of circular RNA METTL9 (circMETTL9) in rat cerebral cortex following traumatic brain injury (TBI). **A** Clustered heat map showing circRNAs differentially expressed in the cortex of TBI model rats compared to sham controls according to microarray analysis. **B** Volcano map showing the 74 circRNAs significantly upregulated and 68 circRNAs significantly downregulated by TBI. **C** KEGG pathway analysis suggesting that these differentially expressed circRNAs may regulate secondary injury following TBI via multiple signaling pathways. **D** GO analysis showing the possible functions of these differentially expressed circRNAs. **E** Sequencing results of seven selected circRNAs with differential expression. **F** qPCR validation of these seven selected circRNAs. *P < 0.05, **P < 0.01, ***P < 0.001 by Student’s* t*-test. **G** Four human circRNAs originating from the METTL9 gene with high homology to rat circMETTL9 according to BLAST searching of circBase. **H** Flowchart of circRNA screening for possible regulation by TBI

To investigate the potential involvement of circRNAs in neuropathology following TBI, four TBI and four sham group cortical tissue samples were analyzed for circRNA expression profiles using a high-throughput circRNA microarray. A total of 143 circRNAs (Additional file 6: Table S6) were differentially expressed between groups (74 upregulated and 68 downregulated in TBI samples) (Fig. 1A, B). Gene ontogeny (GO) and Kyoto Encyclopedia of Genes and Genomes (KEGG) enrichment analyses of these differentially expressed circRNAs suggested involvement in secondary injury following TBI (Fig. 1C, D). Of these differentially expressed circRNAs, 14 were selected for further analyses due to the dramatic difference in expression or high level of expression, but only seven could be amplified by specific primers (Additional file 7: Table S7). While we did verify the expression of these seven circRNAs by qPCR (Fig. 1F, H), only three circRNAs (circMETTL9, circMED13, circUBN2) in Fig. 1F showed the same trend with sequencing results in Fig. 1E. Among them, circMETTL9 exhibited the highest expression level and the most significant difference between TBI and sham groups (Fig. 1F). Additionally, the full-length sequence of circMETTL9 showed high homology with 4 human circRNAs originating from the gene encoding METTL9 according to BLAST analysis (Fig. 1G). These data suggest that the biogenesis of circMETTL9 is conserved in mammals.

### Characterization of circMETTL9 in TBI model rats

**Fig. 2:**
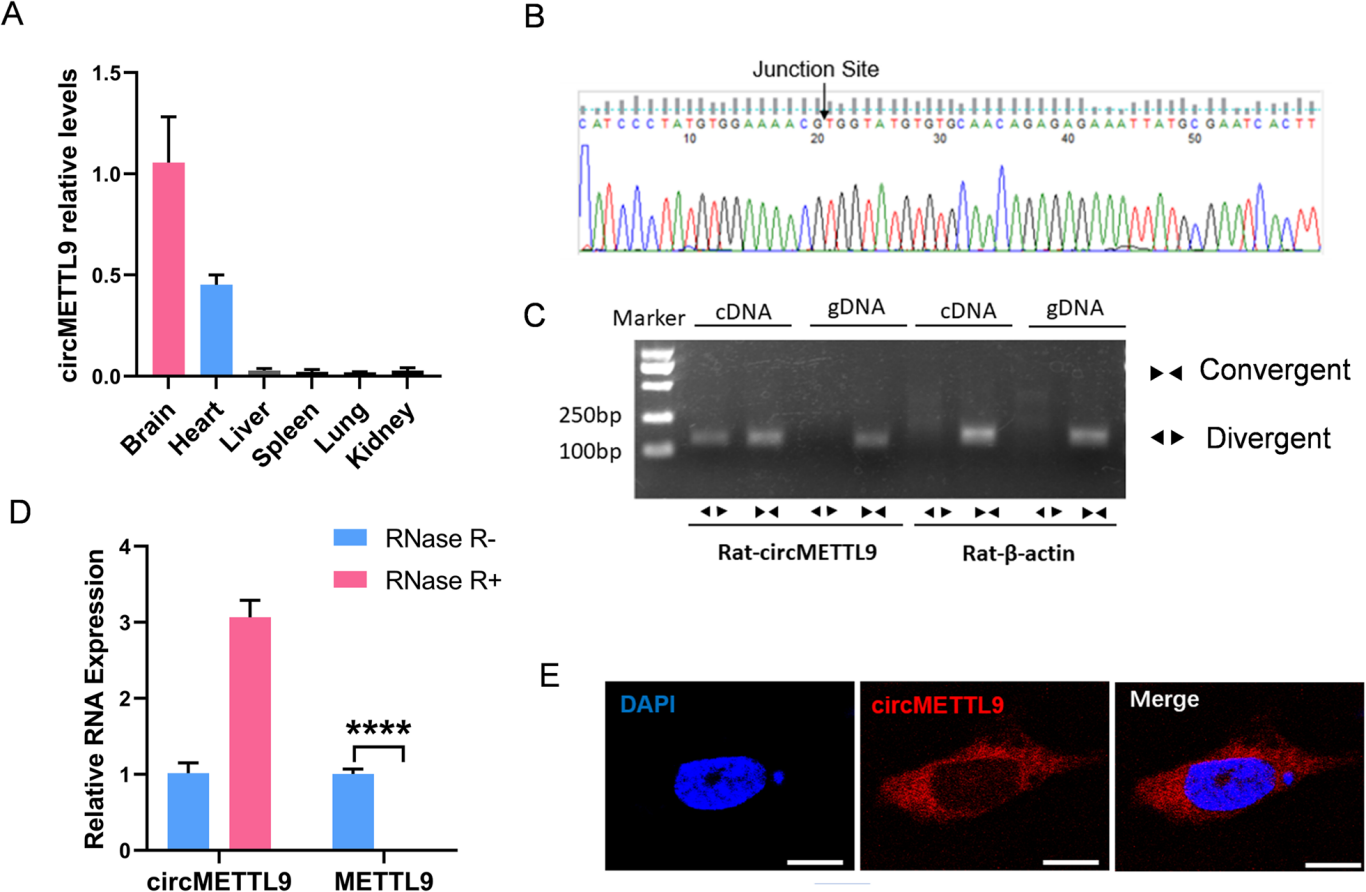
Characterization of rat circMETTL9. **A** Measurement of relative circMETTL9 express in multiple tissues by qPCR (brain, heart, liver, spleen, lung, and kidney, *n* = 3 per group) showing brain enrichment. **B** Sanger sequencing showing the spliced junction of circMETTL9. **C** Presence of circMETTL9 in rat cerebral cortex validated by RT-PCR and agarose gel electrophoresis. CircMETTL9 was amplified by divergent primers from cDNA but not from gDNA. **D** Expression of circMETTL9 and METTL9 in rat cortex after treatment with RNase R as measured by qPCR (*n* = 3 per group). Data presented as mean ± SEM, ****P < 0.0001 by Student’s* t*-test. **E** Localization of circMETTL9 in astrocytes by RNA fluorescence in situ hybridization (FISH). Nuclei were counterstained with DAPI. Scale bar = 10 µm

Examination of circMETTL9 expression levels in multiple organs (including heart, liver, lung, spleen, and kidney) revealed enrichment in brain (Fig. 2A). We then confirmed the circular structure of circMETTL9 by Sanger sequencing (Fig. 2B), RT-PCR and agarose gel electrophoresis (Fig. 2C), and RNase R treatment (Fig. 2D). Sanger sequencing identified the splice sites of circMETTL9 (Fig. 2B), while RT-PCR and agarose gel electrophoresis revealed that divergent primers could amplify circMETTL9 from cDNA but not gDNA (Fig. 2C), and RNase R treatment confirmed that circMETTL9 was resistant to nuclease digestion (Fig. 2D). These findings indicate that circMETTL9 indeed has a circular structure. Furthermore, FISH revealed that circMETTL9 was mainly localized to the cytoplasm of astrocytes, with little detectable expression in the nucleus (Fig. 2E). Collectively, these results demonstrate that circMETTL9 is a conserved, abundant, and stable circRNA differentially upregulated in rat cortical astrocytes following TBI.

### Knockdown of circMETTL9 in the injured cortex of TBI model rats accelerates functional recovery and reduces nerve cell apoptosis

**Fig. 3:**
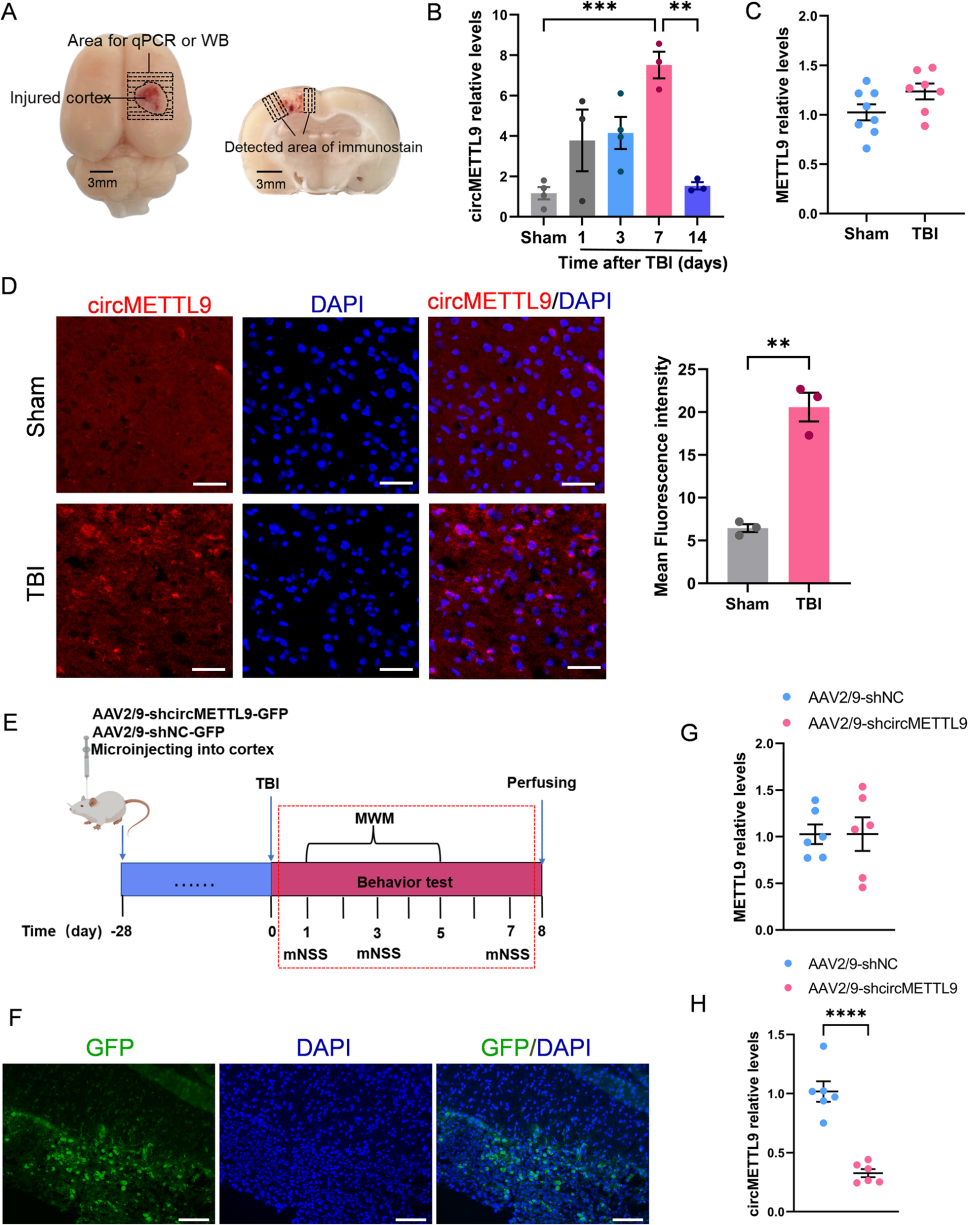
Elevated expression of circMETTL9 in cortex following TBI and successful siRNA-mediated knockdown. **A** Gross observation of brain tissue in TBI rats. Left panel: shaded areas illustrate the injured cortex that was harvested for qPCR and WB analysis. Right panel: the microphotographed areas used in IF, FISH, and TUNEL. Scale bar = 3 mm. **B** Expression levels of circMETTL9 in cortex on days 1, 3, 7, and 14 post-TBI as measured by qPCR. Expression peaked on day 7. **P < 0.01 and ***P < 0.001 by one-way ANOVA. **C** Expression of METTL9 mRNA in cortex of TBI model rats vs. sham controls. **D** Left panel: FISH image indicating that circMETTL9 (red) was highly expressed at the injury site of TBI model rats. Scale bar = 50 µm. Right panel: quantification of mean fluorescence intensity of circMETTL9 (*n* = 3 per group). **P < 0.01 by Student’s* t*-test. **E** Experimental procedure and timeline for in situ knockdown experiments. **F** Representative images of rat cerebral cortex microinjected with AAV2/9 virus expressing siRNA and GFP. GFP expression was measured 4 weeks after microinjection as an index of viral vector expression. Scale bar = 100 µm. **G**, **H** Expression levels of circMETTL9 and METTL9 mRNA 4 weeks after microinjection as measured by qPCR. Expression of circMETTL9 was significantly downregulated while METTL9 mRNA was not significantly altered (*n* = 6 per group). ****P < 0.0001 by Student’s* t-*test

**Fig. 4:**
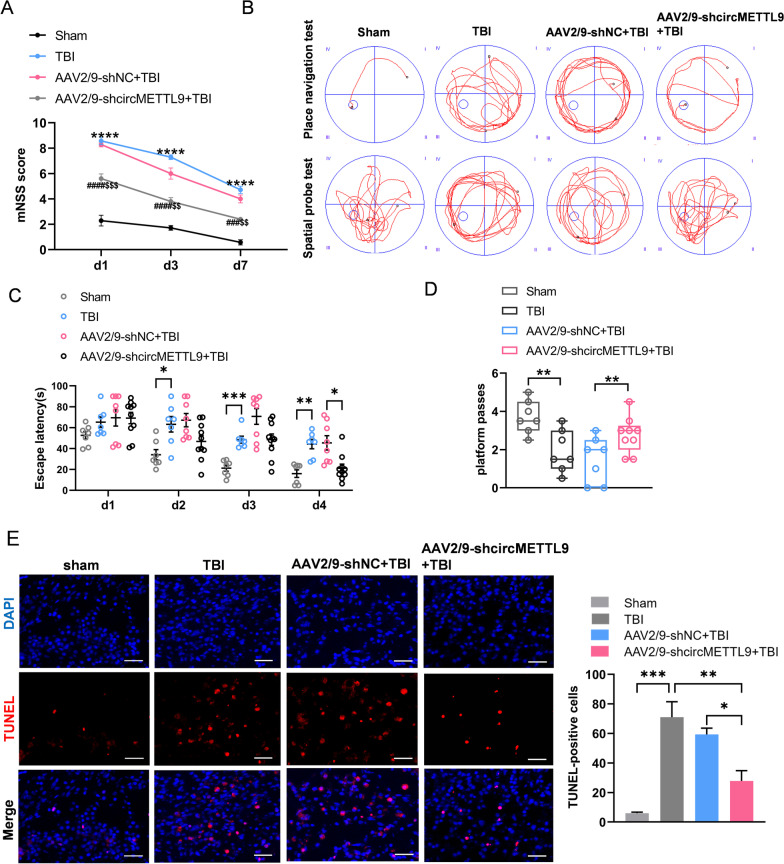
Knockdown of circMETTL9 ameliorates neurological dysfunction, cognitive impairment, and nerve cell apoptosis Induced by TBI. **A** Knockdown of circMETTL9 accelerates functional recovery according to mNSS evaluation on days 1, 3, and 7 post-TBI (Sham, *n* = 7; TBI, *n* = 7; AAV2/9-shNC + TBI, *n* = 8; AAV2/9-shcircMETTL9 + TBI, *n* = 10). ****P < 0.0001 vs. the sham group; ^###^*P* < 0.001 and ^####^*P* < 0.0001 vs. TBI group; ^$$^*P* < 0.01 and ^$$$^*P* < 0.001 compared to the AAV2/9-shNC group (all by two-way repeated measures ANOVA). **B**–**D** Knockdown of circMETTL9 improves spatial learning and memory after TBI. **B** Typical swimming paths in the Morris water maze during the training trials and probe trial. **C** Escape latencies to find a hidden platform (an index of spatial learning). *P < 0.05, **P < 0.01 and ***P < 0.001 by two-way repeated measures ANOVA. **D** Number of platform crossings (an indicator of spatial memory) (Sham, *n* = 7; TBI, *n* = 7; AAV2/9-shNC + TBI, *n* = 8; AAV2/9-shcircMETTL9 + TBI, *n* = 10). **P < 0.01 by one-way ANOVA. **E** Left panel: TUNEL staining showing that circMETTL9 knockdown ameliorates nerve cell apoptosis on the 7th day post-TBI. Scale bar = 50 µm. Right panel: Quantification of relative apoptosis rate (*n* = 3 per group). *P < 0.05, **P < 0.01, and ***P < 0.001 by one-way ANOVA

To investigate if circMETTL9 contributes to secondary neurodegeneration, loss of function, or recovery from TBI, we established TBI model with controlled cortical injury in rats (Fig. 3A). We first assessed the expression levels of circMETTL9 on days 1, 3, 7, and 14 post-TBI by qPCR, and found that expression was highest on the 7th day (Fig. 3B). Surprisingly, there was no significant difference in brain METTL9 mRNA expression between sham and TBI group rats on the 7th day (Fig. 3C). Furthermore, FISH of brain sections isolated 7 days post-TBI confirmed high expression of circMETTL9 at the site of traumatic injury (Fig. 3D). To test whether this upregulation of circMETTL9 contributed to secondary injury, we compared functional recovery between model rats microinjected with vehicle or AAV2/9-shcircMETTL9 at the injury site in right frontal cortex (Fig. 3E). Expression of shcircMETTL9 was first confirmed by examining emission of the vector marker green fluorescent protein (GFP) 4 weeks post-injection (Fig. 3F). At this time, circMETTL9 was indeed significantly downregulated, while METTL9 mRNA expression was unchanged (Fig. 3G, H). Knockdown of circMETTL9 expression following TBI accelerated functional recovery as revealed by the mNSS (Fig. 4A). Further, knockdown improved spatial learning as indicated by reduced escape latencies in the training phase of the Morris water maze test compared to vehicle-treated TBI model rats (Fig. 4B–D) and enhanced spatial memory as indicated by a greater number of former platform crossings in the probe test. Consistent with improved spatial learning and memory, circMETTL9 knockdown also reduced the number of apoptotic nerve cells at the injury site as evidenced by TUNEL staining (Fig. 4E). Collectively, these results suggest that circMETTL9 contributes to neurodegeneration and loss of neurological function following TBI, and that downregulation of circMETTL9 can prevent secondary neurodegeneration and concomitant neurological deficits.

### CircMETTL9 promoted LPS-induced inflammatory response in astrocytes

**Fig. 5:**
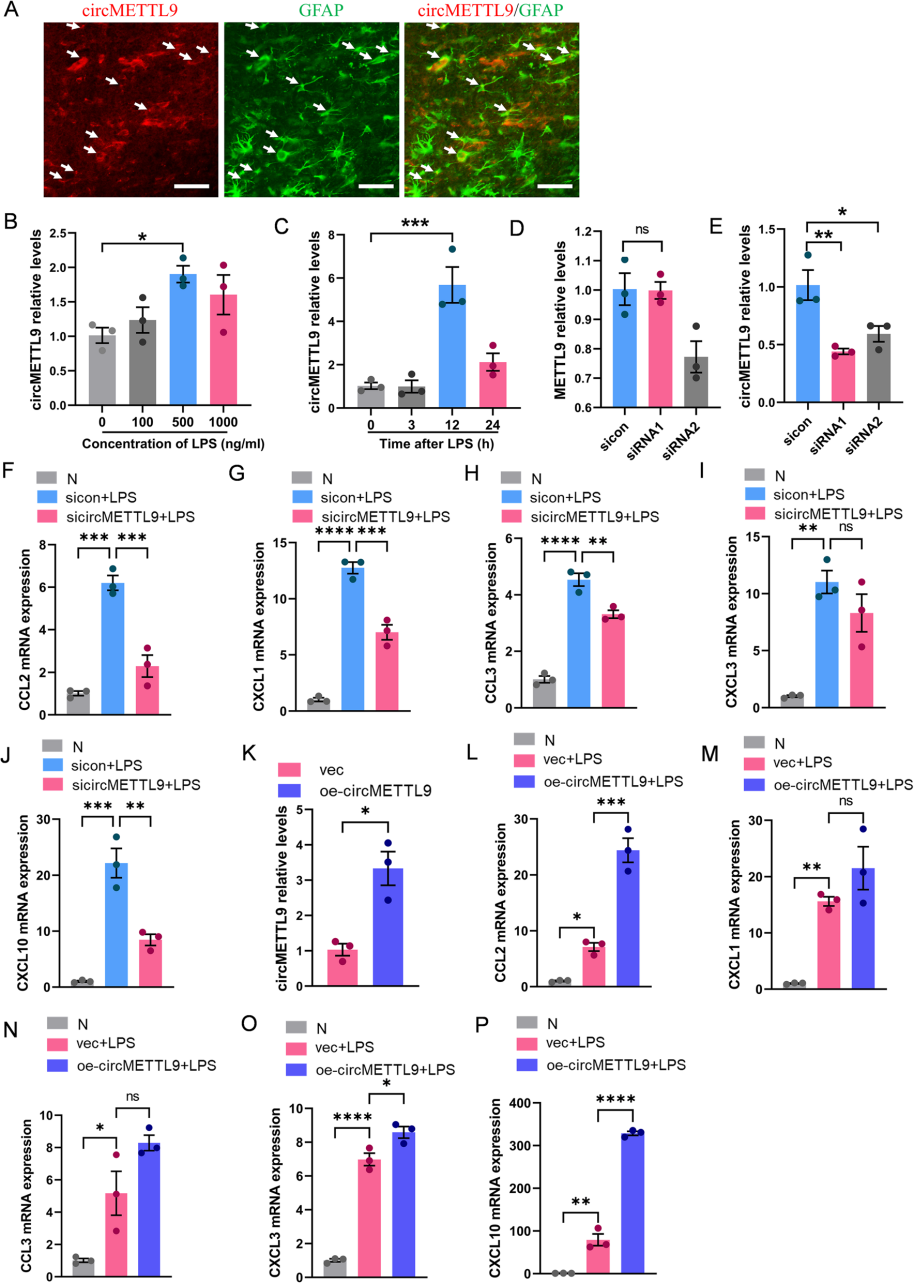
Upregulation of circMETTL9 in cultured astrocytes promotes inflammatory signaling. **A** Representative image showing co-localization of the astrocyte marker GFAP (green) and circMETTL9 (red) in rat cerebral cortex. White arrows show the typical co-localization of circMETTL9 and GFAP in the injured cortex. Scale bar = 50 µm. **B** LPS-induced upregulation of circMETTL9 in cultured astrocytes as measured by qPCR. *P < 0.05 by one-way ANOVA. **C** Peak expression after a 12-h treatment with 500 ng/mL LPS. ***P < 0.001 by one-way ANOVA. **D**, **E** Design of siRNA sequences for circMETTL9 knockdown and detection of circMETTL9 and METTL9 mRNA expression levels following transfection for 48 h using Lipo3000. *P < 0.05 and **P < 0.01 by Student’s *t*-test. **F**–**J** Changes in the astrocytic expression levels of pro-inflammatory cytokines CCL2, CXCL1, CCL3, CXCL3, and CXCL10 after circMETTL9 knockdown. **P < 0.01, ***P < 0.001, and ****P < 0.0001 by one-way ANOVA. (K) CircMETTL9 is significantly upregulated after 48 h of plasmid transfection. *P < 0.05, Student’s* t*-test. **L**–**P** Changes in expression levels of CCL2, CXCL1, CCL3, CXCL3, and CXCL10 in astrocytes overexpressing circMETTL9 compared to controls. *P < 0.05, **P < 0.01, ***P < 0.001, and ****P < 0.0001 by one-way ANOVA

In our previous study, we reported that secondary degeneration following TBI is strongly dependent on neuroinflammatory signaling by astrocytes [20], so we examined if circMETTL9 regulates astrocytic inflammatory signaling. To this end, we first examined the cellular distribution of circMETTL9 by dual immunofluorescence staining and found strong co-expression with the astrocyte-specific marker GFAP in the injured cortex (Fig. 5A). Next, to examine circMETTL9 regulation of astrocytic inflammatory signaling in isolation, we utilized cultured primary astrocytes activated by the pro-inflammatory factor LPS. Exposure to 500 ng/mL LPS for 12 h markedly upregulated circMETTL9 expression (Fig. 5B, C), suggesting circMETTL9 involvement in neuroinflammation. To further examine the effects of circMETTL9 expression on inflammatory signaling, we designed two siRNA sequences (siRNA1 and siRNA2) for circMETTL9 knockdown and found that transfection with siRNA1 dramatically reduced the expression of circMETTL9 without altering expression of the source gene METTL9 (Fig. 5D, E). Furthermore, knockdown of circMETTL9 led to significant downregulation of pro-inflammatory cytokines CCL2, CXCL1, CCL3, and CXCL10 (but not CXCL3) in cultured astrocytes (Fig. 5F–J). Conversely, transfection with a plasmid encoding circMETTL9 significantly upregulated the expression of circMETTL9 (Fig. 5K) and concomitantly induced significant elevations in the expression levels of CCL2, CXCL3, and CXCL10 compared empty vector-transfected control cultures (Fig. 5L, O, P). In addition, the expression levels of CXCL1 and CCL3 were also upregulated but the difference from controls did not reach statistical significance (Fig. 5M, N). These data strongly suggest that circMETTL9 is involved in the inflammatory response of astrocytes following TBI.

### The circMETTL9 interacts with SND1 protein in astrocytes

**Fig. 6:**
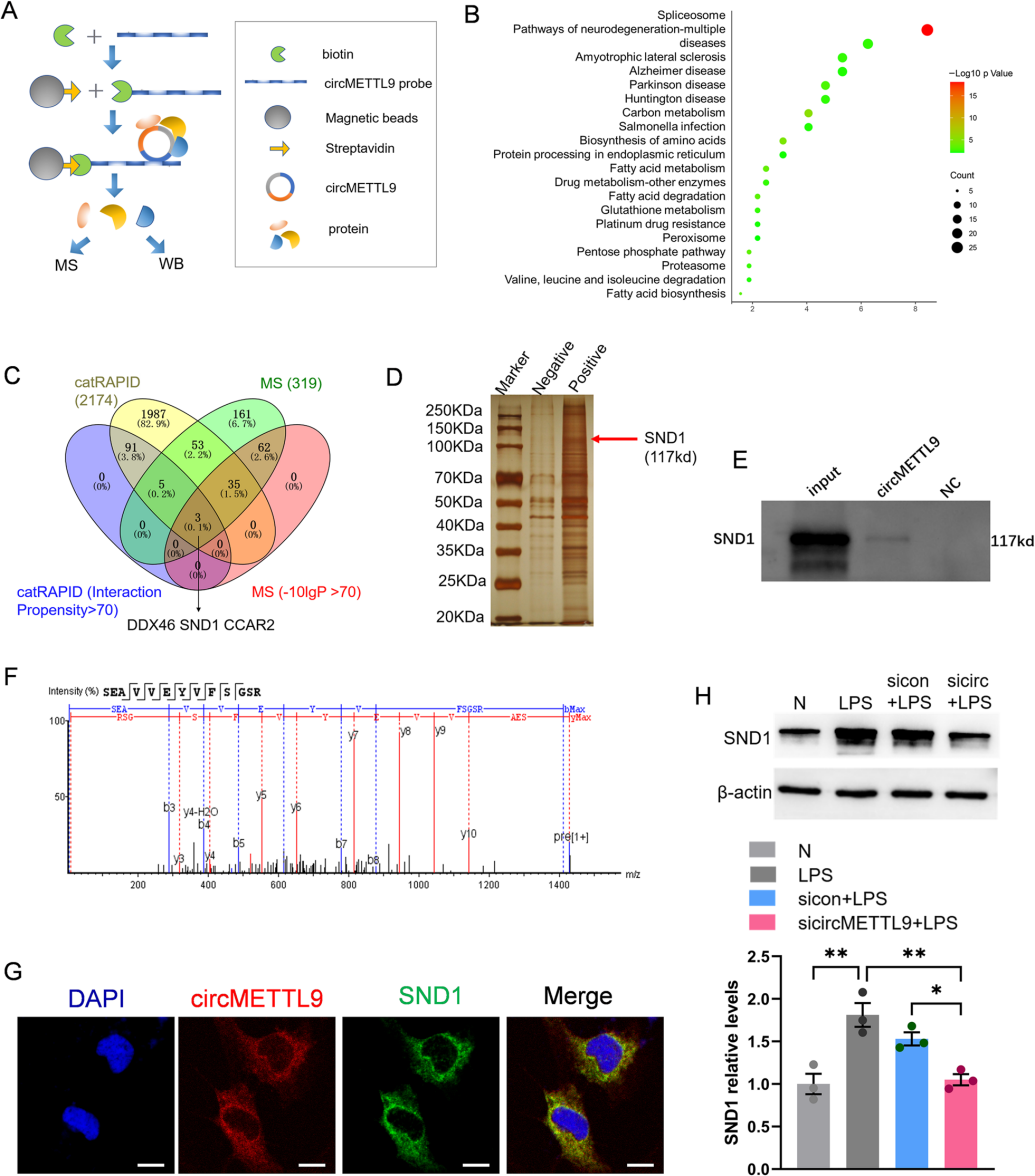
CircMETTL9 interacts with SND1 protein in astrocytes. **A** Experimental procedure for protein pull-down by circMETTL9. First, circMETTL9 probe is biotinylated and added to lysate from LPS-stimulated astrocytes. Magnetic beads with bound streptavidin are then added to capture biotinylated probe and associated bound proteins. Finally, proteins are obtained by washing the magnetic beads. **B** KEGG pathway analysis of the top twenty enriched proteins bound to circMETTL9 according to mass spectrometry. **C** Overlap of proteins in catRAPID with Interaction Propensity > 70 and proteins with -10lgP > 70 by mass spectrometry (potential circMETTL9-interacting proteins). **D** Silver staining showing the presence of SND1. **E** Detection of SND1 by western blot after pull-down from astrocyte lysate by circMETTL9 probe but not negative probe. **F** Ion peak pattern showing the SND1 peptide, it is the secondary map of SND1 protein to a specific peptide. **G** Co-localization of SND1 (green) with circMETTL9 (red) in astrocyte cytoplasm by combined IF-FISH. Scale bar = 50 µm. **H** Representative western blots of SND1 expression in astrocytes after knockdown of circMETTL9. *P < 0.05, **P < 0.01, and ***P < 0.001 by one-way ANOVA

We next examined potential molecular mechanisms mediating circMETTL9-induced inflammatory signaling in astrocytes. As exonic circRNAs in the cytoplasm generally act as miRNA or protein sponges, we first screened for possible circMETTL9-binding miRNAs from miRbase using miRanda software, but identified only four potential miRNAs sponging targets (Additional file 8: Table S8). Alternatively, we identified 2175 proteins that may bind circMETTL9 according to the catRAPID algorithm (Additional file 9: Table S9), suggesting that circMETTL9 may function as an RNA-binding protein (RBP) sponge. To test this notion, we performed RNA pull-down assays and mass spectrometry (Fig. 6A). Indeed, a total of 319 proteins were pulled-down by the circMETTL9 probe (Additional file 10: Table S10), and KEGG pathway analysis suggested that most of these proteins are associated with neurodegeneration and central nervous system diseases (Fig. 6B).

To examine if any of these proteins could actually interact with circMETTL9 and influence inflammation, we first investigated the overlap between proteins in catRAPID with Interaction Propensity > 70 and proteins with -10lgP > 70 in mass spectrometry, which yielded three proteins (DDX46, SND1, CCAR2) (Fig. 6C). We also found that the amino acid sequence of SND1 shared 98–99% identity with rat NF-κB, a central transcription factor controlling inflammatory pathways [21, 22]. Therefore, we hypothesized that SND1 may interact with circMETTL9 in astrocytes to regulate inflammatory signaling.

We first screened for a direct interaction of circMETTL9 with SND1 by RNA pull-down (Fig. 6D, E). The ion peak pattern confirmed the presence of SND1 among proteins pulled-down by circMETTL9 probe (Fig. 6F), while combined IF-FISH revealed co-localization of circMETTL9 and SND1 in astrocyte cytoplasm (Fig. 6G). Moreover, SND1 was upregulated in LPS-stimulated astrocytes, a response reversed by circMETTL9 knockdown (Fig. 6H). These data indicate that circMETTL9 can interact with and promote the expression of SND1.

### CircMETTL9 may promote the LPS-induced inflammatory response in astrocytes by interacting with SND1

**Fig. 7:**
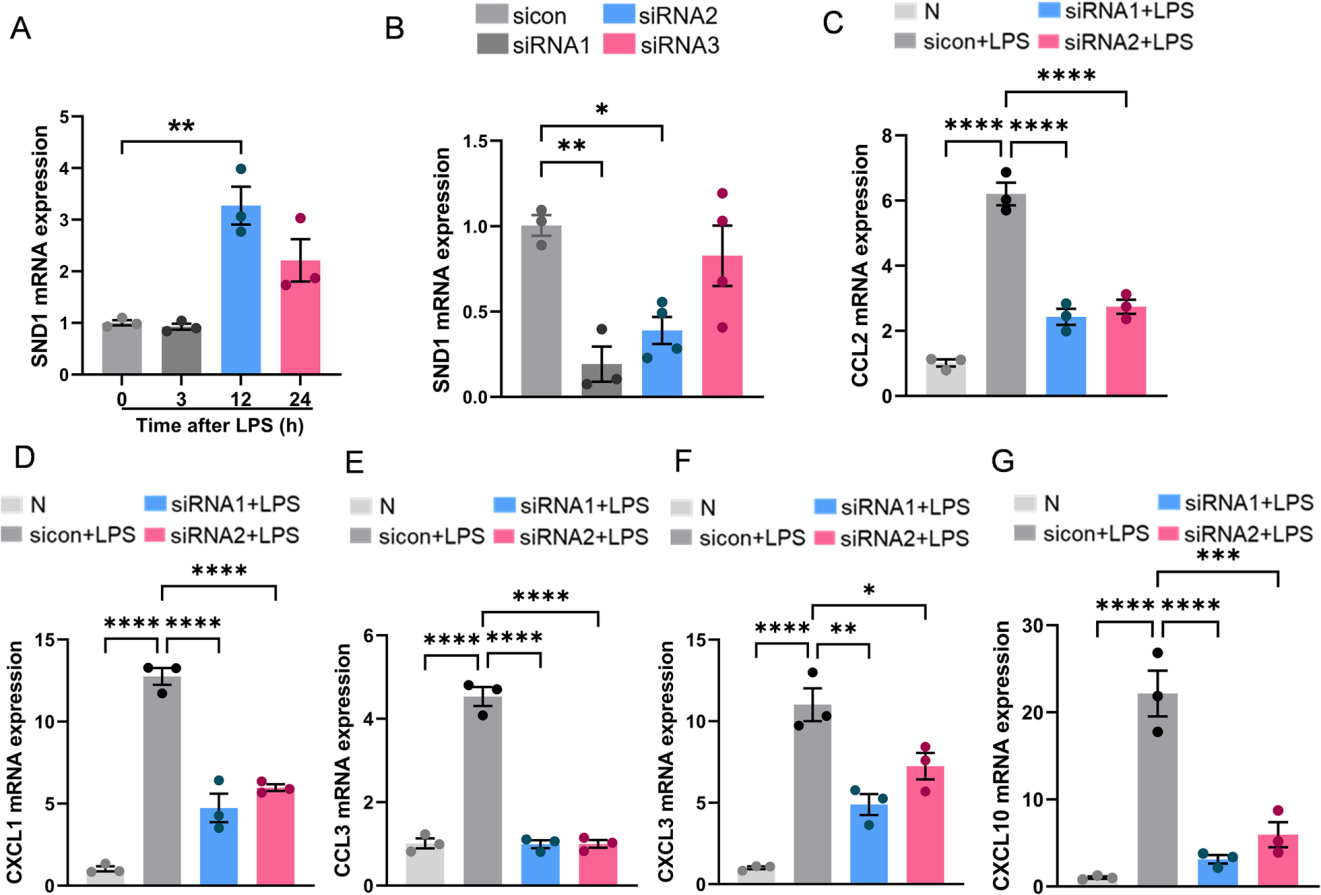
SND1 mediates the LPS-induced inflammatory response in astrocytes. **A** Treatment of astrocytes with 500 ng/mL LPS for 3, 12, and 24 h elevated SND1 expression as measured by qPCR. **P < 0.01 by one-way ANOVA. **B** Two of three siRNAs targeting SND1 successfully knocked down SND1 in astrocytes after transfection. *P < 0.05 and **P < 0.01 by one-way ANOVA. **C**–**G** Astrocyte expression levels of CCL2, CXCL1, CCL3, CXCL3, and CXCL10 were elevated by LPS, while 2 different siRNAs reversed these effects. *P < 0.05, **P < 0.01, ***P < 0.001, and ****P < 0.0001 by one-way ANOVA

We next examined SND1 functions in the LPS-induced inflammatory response of astrocytes. Treatment of cultured astrocytes with LPS for 3, 12, and 24 h upregulated SND1 mRNA compared to untreated controls, with the greatest upregulation at 12 h (Fig. 7A). As expected, this enhancement was inhibited by two SND1 siRNAs (Fig. 7B). Furthermore, these siRNAs reversed the LPS-induced upregulation of CCL2, CXCL1, CCL3, CXCL3, and CXCL10 in astrocytes, indicating that SND1 promoted the LPS-induced inflammatory response in astrocytes (Fig. 7C–G).

**Fig. 8:**
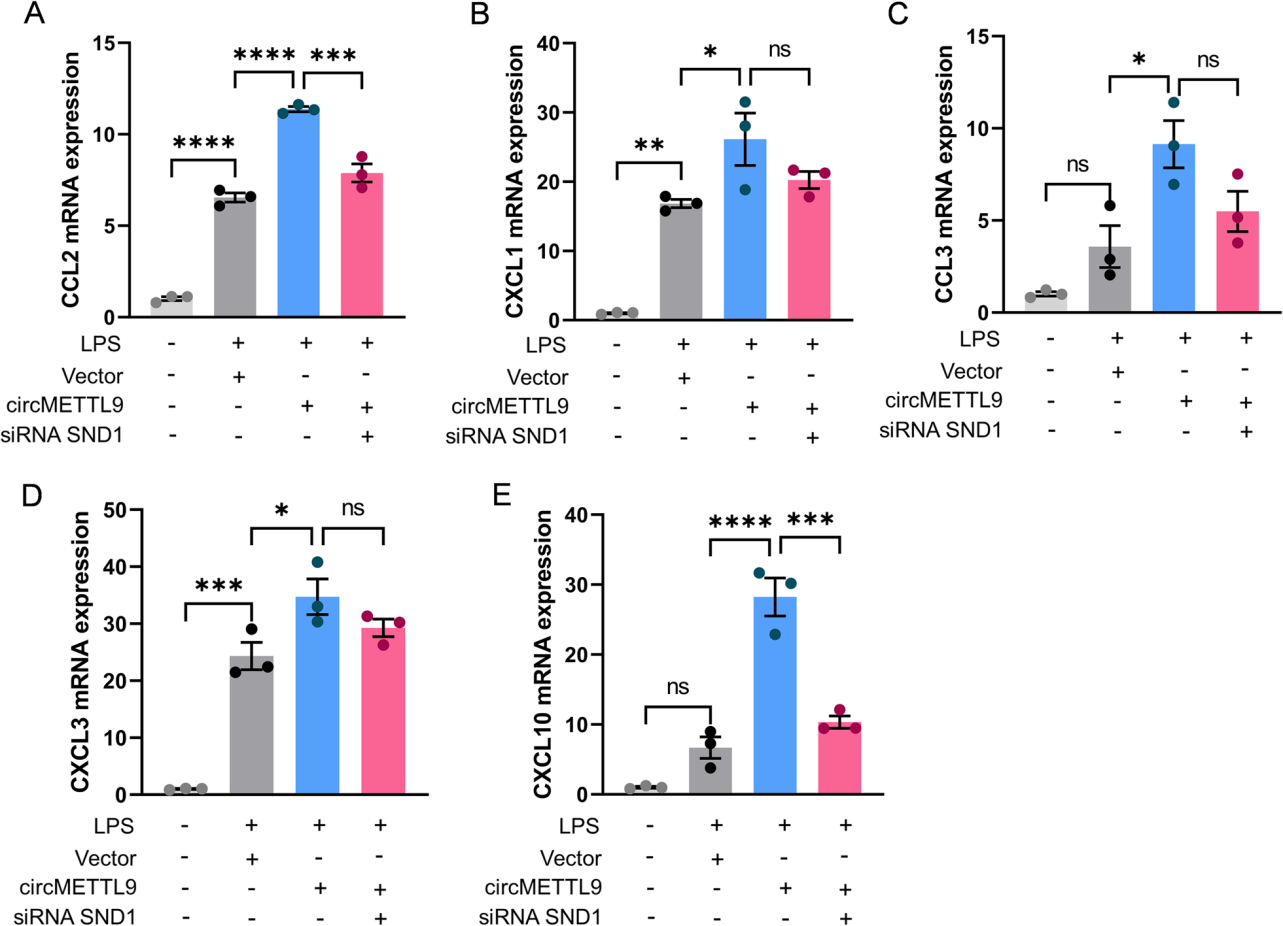
SND1 is regulated by circMETTL9. **A**–**E** Astrocytes transfected with circMETTL9 overexpression plasmid demonstrated stronger LPS-induced inflammatory responses than astrocytes co-transfected with circMETTL9 overexpression plasmid and an SND1-targeted siRNA as evidenced by expression levels of CCL2, CXCL1, CCL3, CXCL3, and CXCL10. *P < 0.05, **P < 0.01, ***P < 0.001, and ****P < 0.0001 by one-way ANOVA

Conversely, overexpression of exogenous circMETTL9 upregulated CCL2, CXCL1, CCL3, CXCL3, and CXCL10 expression levels in the presence of LPS stimulation. However, upregulation of these chemokines by circMETTL9 overexpression was reversed by co-transfection of oe-circMETTL9 (overexpression of exogenous circMETTL9) and si-SND1 (knockdown of SND1) (Fig. 8A–E). These results and those presented in section  “The circMETTL9 interacts with SND1 protein in astrocytes” strongly suggest that circMETTL9 promotes the LPS-triggered inflammatory response by interacting with SND1.

### Knockdown of circMETTL9 may also mitigate cortical neuroinflammation in TBI model rats by downregulating SND1

**Fig. 9:**
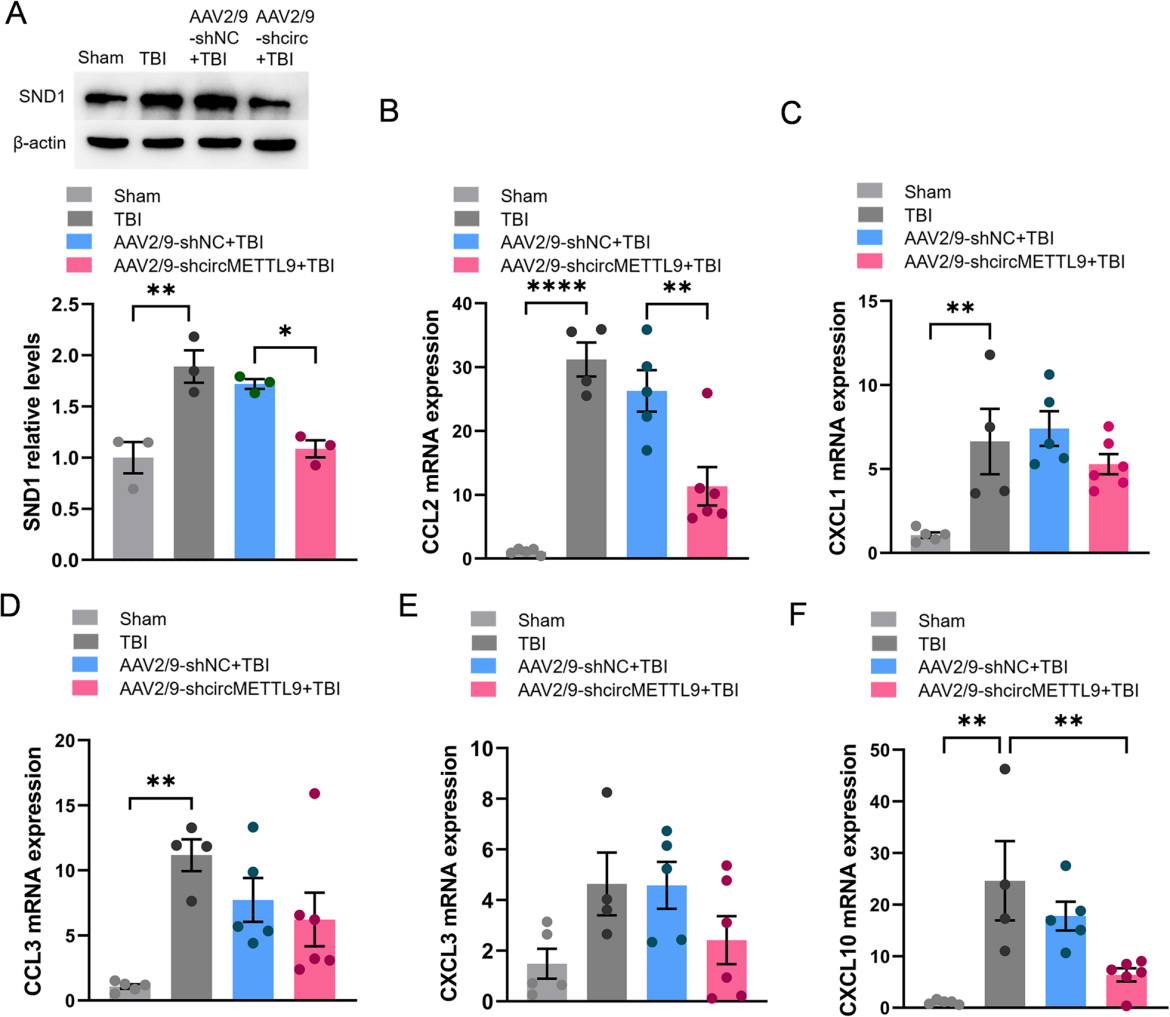
Knockdown of circMETTL9 mitigates cortical neuroinflammation in TBI model rats by downregulating SND1. **A** Representative western blots of SND1 expression in the cerebral cortex of rats 7 days post-TBI. *P < 0.05 and **P < 0.01 by one-way ANOVA. **B**–**F** Expression levels of CCL2, CXCL1, CCL3, CXCL3, and CXCL10 in cortex 7 days post-TBI as measured by qPCR. *P < 0.05, **P < 0.01, ***P < 0.001, and ****P < 0.0001 by one-way ANOVA

Finally, we examined whether knockdown of circMETTL9 also downregulates SND1 expression and attenuates neuroinflammation following TBI. Expression of SND1 was upregulated at the injury site of TBI model rats, and this response was reversed by circMETTL9 knockdown (Fig. 9A). Additionally, the expression levels of CCL2, CXCL1, CCL3, CXCL3, and CXCL10 were upregulated in TBI rat cortex, and knockdown of circMETTL9 significantly reversed elevated CCL2 expression (Fig. 9B), while expression levels of CXCL1, CCL3, CXCL3, and CXCL10 were also reduced but not to the level of statistical significance (Fig. 9C–F).

## Discussion

Recent developments in next-generation sequencing and bioinformatics have provided evidence that circRNAs are important functional regulators of gene expression and not merely by-products of mis-splicing or transcriptional noise [7]. Using circRNA chip screening, we identified a number of circRNAs differentially expressed between TBI model rats and sham-treated controls, of which circMETTL9 demonstrated among the greatest expression. However, differential expression was examined only at a single time point (12-h post-TBI) and the logarithmic fold-change in circMETTL9 was only 0.86, suggesting that other circRNAs may also be significantly up- or down-regulated by TBI and contribute to further brain injury or conversely to functional recovery. Nonetheless, we also found that circMETTL9 was still upregulated several days post-TBI and peaked on the seventh day post-TBI, was specifically distributed at the injury site, and was highly conserved, underscoring its potential importance in secondary injury after TBI. Indeed, inhibition of circMETTL9 significantly attenuated neurological and cognitive dysfunction in TBI model rats and reduced inflammatory signaling in astroglial cells. This is the first study to demonstrate the functional involvement of a cerebral cortex-specific and conserved circRNA, circMETTL9, in the regulation of TBI-induced neuroinflammation and secondary injury.

Astrocytes respond to local tissue damage by releasing a variety of chemokines, cytokines, and growth factors that ultimately determine the extent of neuroinflammatory damage and the efficacy of endogenous repair [23]. A large number of clinical and animal studies have shown that upregulation of pro-inflammatory chemokines after TBI is strongly associated with neurological dysfunction and that inhibition of pro-inflammatory chemokine signaling can improve functional recovery [23–26]. Furthermore, in our previous studies, we found that upregulation of CCL2, CXCL1, and CXCL10 in astrocytes aggravated secondary injury and neurological dysfunction in TBI model rats, while inhibitors of NF-κB, JNK, ERK, and p38 significantly suppressed both CCL2 and CXCL1 expression and promoted functional recovery following TBI [17, 27–29]. In the present study, we found that circMETTL9 co-localized with astrocytes in cerebral cortex, was upregulated in LPS-treated astrocytes, and potentiated pro-inflammatory cytokine expression by astrocytes, strongly suggesting that circMETTL9 exacerbates the neuroinflammatory response following TBI by enhancing pro-inflammatory signaling in astrocytes.

Circular RNAs have been shown to regulate gene expression through several molecular mechanisms [30–32]. Many circRNAs regulate the expression of specific mRNAs by sponging, which in turn may contribute to the pathogenesis and development of TBI [33–35]. Herein, we present evidence for a novel mechanism by which circMETTL9 regulates chemokine expression and signaling, namely through formation of a ternary complex with SND1, a multifunctional protein with 98%–99% homology to the inflammation-associated transcription factor NF-κB (p105) in rat and NF-κB2 (p100) in human [22]. SND1 is an N6-methyladenosine (m^6^A) reader, and several studies have shown that the m^6^A site of circRNA can bind to the corresponding m^6^A reader and participate in regulation of RNA processing, translation, and stability [36–38]. For example, upregulated circNSUN2 (derived from NSUN2, another RNA methyltransferase with m^6^A sites) can be combined with multiple m^6^A readers in colorectal cancer cells [10]. The source gene of circMETTL9, METTL9, is also an RNA methyltransferase [39, 40], and analysis using SRAMP suggested that circMETTL9 may have multiple m^6^A sites. Therefore, we speculate that SND1 and circMETTL9 are highly likely to bind in situ as in our pull-down assays. Whether this binding is mediated by m^6^A warrants further study.

Next, we demonstrated that circMETTL9 knockdown decreased the levels of SND1 protein and that SND1 regulated the expression of multiple chemokines in astrocytes. Several studies have shown that SND1 can regulate inflammation by activating NF-κB [41–43]. Furthermore, we recently reported that NF-κB inhibitors decreased the upregulation of CCL2 and CXCL1 following TBI [17]. Moreover, SND1 has been implicated in post-transcriptional regulation, including pre-mRNA splicing and spliceosome assembly [44] as well as RNA interference, stability, and editing [45–47]. We speculated that certain mRNAs important to TBI outcome interact with SND1, and found that CCL2, CCL3, and CXCL3 mRNAs exhibited high binding probability according to the catRAPID algorithm. Moreover, SND1 knockdown reduced LPS-induced upregulation of CCL2, CXCL1, CCL3, and CXCL3 in astrocytes. Thus, SND1 may regulate neuroinflammation of LPS-stimulated astrocytes by directly binding and stabilizing CCL2, CCL3, and CXCL3 mRNAs.

Consistent with our finding that inflammation can be regulated through SND1 binding to non-coding RNA, it was reported that LincRNA TINCR facilitates inflammation in post-burn skin fibroblasts by directly binding to SND1 and inducing TGF-β1 expression [48]. In addition, the circ_0004087/SND1/MYB/BUB1 axis was reported to modulate the error mitosis correction mechanism in prostate cancer cells [49]. However, the specific mechanisms underlying SND1 protein upregulation by circMETTL9 interaction are still unclear. It was previously reported that circECE1 interacts with C-Myc and inhibits C-Myc degradation by preventing speckle-type POZ-mediated C-Myc ubiquitination. By analogy, formation of the circMETTL9-SND1 complex may prevent SND1 degradation, a possibility that warrants further study.

This study had several limitations. First, we did not examine the localization of circMETTL9 in other cell types. While circMETTL9 was localized to injured cortical astrocytes and significantly upregulated in cultured astrocytes treated with the pro-inflammatory factor LPS, it is still possible that circMETTL9 may also regulate inflammatory signaling in neurons and microglia, an issue that we will addressed in future studies. Second, not all chemokines upregulated by TBI or LPS treatment were downregulated by circMETTL9 knockdown, suggesting that there may be other inflammatory regulators controlling expression as well as other potential targets under regulatory control by circMETTL9. Third, while LPS is not ideal to mimic TBI-specific scenarios in vitro, it currently is an accepted proof-of-concept study. Finally, further studies are needed to determine whether circMETTL9 is a feasible target for clinical intervention.

## Conclusions

The highly conserved circular RNA circMETTL9 was abundantly expressed in the astrocytes of rat brain and markedly upregulated following TBI. Downregulation of circMETTL9 reduced neuroinflammatory signaling, nerve cell apoptosis, and neurological dysfunction, including spatial learning and memory impairments, in TBI model rats. CircMETTL9 binds SND1 and upregulates SND1 expression, possibly by protecting it from degradation. Elevated expression of SND1 in turn leads to the upregulation of pro-inflammatory chemokines in astrocytes that drive neuroinflammation and neurodegeneration. To the best of our knowledge, this is the first study to identify a circRNA, namely circMETTL9, that contributes to the secondary damage following TBI.

## Supplementary Information

**Additional file 1: Table S1.** The sequences of siRNAs.

**Additional file 2: Table S2.** The sequence of plasmid to overexpression of circMETTL9.

**Additional file 3: Table S3.** The probe sequences of circMETTL9 for RNA pulldown.

**Additional file 4: Table S4.** The probe sequences of circMETTL9 for FISH.

**Additional file 5: Table S5.** Primer sequences for quantitative real-time PCR (5′ to 3′).

**Additional file 6: Table S6.** Differentially expressed circRNAs obtained by high-throughput circRNA microarray.

**Additional file 7: Table S7.** RT-PCR and agarose gel electrophoresis of 7 circRNAs which were amplified by specific primers.

**Additional file 8: Table S8.** MiRNAs that have the potential to bind to circMETTL9.

**Additional file 9: Table S9.** Proteins that may bind circMETTL9 obtained by catRAPID algorithm.

**Additional file 10: Table S10.**  Proteins that may bind circMETTL9 obtained by pulldown assays and mass spectrometry.

## Data Availability

All supporting data presented in the study are included in the article and additional material, further inquiries can be obtained from the corresponding author.
