# Supplementary material for: Circular RNA METTL9 contributes to neuroinflammation following traumatic brain injury by complexing with astrocytic SND1
Source: J Neuroinflammation. 2023 Feb 17;20:39. doi: 10.1186/s12974-023-02716-x (PMC9936775; doi:10.1186/s12974-023-02716-x)
Supplement: Supplementary file 1 — Additional file 1: Table S1. The sequences of siRNAs. [file 12974_2023_2716_MOESM1_ESM.docx]

**Supplementary Table 1. The sequences of siRNAs.**

| Target Gene | Type | siRNA Sequence (5′ to 3′) |
| --- | --- | --- |
| circMETTL9 | siRNA1 | sense: GUGGAAAACGUGGUAUGUGTT |
|  |  | antisense: CACAUACCACGUUUUCCACTT |
|  | siRNA2 | sense: GAAAACGUGGUAUGUGUGCTT |
|  |  | antisense: GCACACAUACCACGUUUUCTT |
| SND1 | siRNA1 | sense: GCAACAUUCGAGCUGGAAATT |
|  |  | antisense: UUUCCAGCUCGAAUGUUGCTT |
|  | siRNA2 | sense: GGAUUAAGUGCCCAACCUUTT |
|  |  | antisense: AAGGUUGGGCACUUAAUCCTT |
|  | siRNA3 | sense: GGGAGACAACAUACAGGAUTT |
|  |  | antisense: AUCCUGUAUGUUGUCUCCCTT |
