# Supplementary material for: Circular RNA METTL9 contributes to neuroinflammation following traumatic brain injury by complexing with astrocytic SND1
Source: J Neuroinflammation. 2023 Feb 17;20:39. doi: 10.1186/s12974-023-02716-x (PMC9936775; doi:10.1186/s12974-023-02716-x)
Supplement: Supplementary file 2 — Additional file 2: Table S2. The sequence of plasmid to overexpression of circMETTL9. [file 12974_2023_2716_MOESM2_ESM.docx]

**Supplementary Table 2. The sequence of plasmid to overexpression of circMETTL9.**

| TGGTATGTGTGCAACAGAGAGAAATTATGCGAATCACTTCAGTCTGTCTTTGTTCAGAGTTATCTTGACCAAGGAACACAGATCTTCTTAAACAACAGCATTGAGAAATCTGGCTGGCTATTTATCCAACTCTATCATTCTTTTGTATCATCTGTTTTTAGCCTGTTTATGTCTAGAACATCTATTAACGGGTTGCTAGGAAGAGGCTCCATGTTTGTGTTCTCACCAGATCAGTTTCAGAGACTGCTTAAAATTAATCCGGACTGGAAAACCCATAGACTTCTTGATTTAGGTGCTGGAGATGGAGAAGTCACGAAAATCATGAGCCCTCATTTTGAAGAAATTTATGCCACTGAACTTTCTGAAACAATGATCTGGCAGCTCCAGAAGAAGAAATACAGAGTGCTTGGTATAAATGAATGGCAGAATACAGGGTTCCAGTATGATGTCATCAGCTGCTTAAATCTGCTGGATCGCTGTGATCAGCCTCTGACATTGTTAAAAGATATCAGAAGTGTCTTGGAGCCCACCCAAGGCAGGGTCATCCTGGCCTTGGTTTTGCCCTTTCATCCCTATGTGGAAAACG |
| --- |
