# Supplementary material for: Circular RNA METTL9 contributes to neuroinflammation following traumatic brain injury by complexing with astrocytic SND1
Source: J Neuroinflammation. 2023 Feb 17;20:39. doi: 10.1186/s12974-023-02716-x (PMC9936775; doi:10.1186/s12974-023-02716-x)
Supplement: Supplementary file 3 — Additional file 3: Table S3. The probe sequences of circMETTL9 for RNA pulldown. [file 12974_2023_2716_MOESM3_ESM.docx]

**Supplementary Table 3. The probe sequences of circMETTL9 for RNA pulldown.**

| Probe Name Sequence |
| --- |
| circMETTL9-Positive1 TCATCCCTATGTGGAAAACGTGGT |
| circMETTL9-Positive2 ATGTGGAAAACGTGGTATGTGTGC |
| circMETTL9-Positive3 AACGTGGTATGTGTGCAACAGAGA |
| circMETTL9-Negative GCACACATACCACGTTTTCCACAT |
