# Supplementary material for: Circular RNA METTL9 contributes to neuroinflammation following traumatic brain injury by complexing with astrocytic SND1
Source: J Neuroinflammation. 2023 Feb 17;20:39. doi: 10.1186/s12974-023-02716-x (PMC9936775; doi:10.1186/s12974-023-02716-x)
Supplement: Supplementary file 4 — Additional file 4: Table S4. The probe sequences of circMETTL9 for FISH. [file 12974_2023_2716_MOESM4_ESM.docx]

**Supplementary Table 4. The probe sequences of circMETTL9 for FISH.**

| Probe Name Sequence |
| --- |
| circMETTL9 5’-TCTCTGTTGCACACATACCACGTTTTCCACATAGGGATGA-3’ |
