# Supplementary material for: Circular RNA METTL9 contributes to neuroinflammation following traumatic brain injury by complexing with astrocytic SND1
Source: J Neuroinflammation. 2023 Feb 17;20:39. doi: 10.1186/s12974-023-02716-x (PMC9936775; doi:10.1186/s12974-023-02716-x)
Supplement: Supplementary file 5 — Additional file 5: Table S5. Primer sequences for quantitative real-time PCR (5′ to 3′). [file 12974_2023_2716_MOESM5_ESM.docx]

**Supplementary Table 5. Primer sequences for quantitative real-time PCR (5′ to 3′).**

| Gene | Species | Forward primer | Reverse primer |
| --- | --- | --- | --- |
| CCL2 | rat | TGCTGCTACTCATTCACTGGC | CCTTATTGGGGTCAGCACAG |
| CXCL1 | rat | AGAACATCCAGAGTTTGAAGGTGAT | GTGGCTATGACTTCGGTTTGG |
| CCL3 | rat | CATGGCGCTCTGGAACGAA | TGCCGTCCATAGGAGAAGCA |
| CXCL3 | rat | GTGCCTGAAGACCCTACCAAG | AGCTTGAGGGTTGAGACAAACT |
| CXCL10 | rat | TTCCGTAAGCTATGTGCAGGTA | TCAGGTGAACTCAGAACTGATG |
| SND1 | rat | GGAGTCCCCTGCCAAAGT | GTGCGAGCGTCCTCATCT |
| β-actin | rat | AGGGAAATCGTGCGTGACAT | GAACCGCTCATTGCCGATAG |
| Has_18s | human | GGAGTATGGTTGCAAAGCTGA | ATCTGTCAATCCTGTCCGTGT |
| circMETTL9 | rat | CATCCCTATGTGGAAAACGTG | TGGATAAATAGCCAGCCAGA |
| Has_  circMETTL9 | human | CATCCCTATGTGGAAAACGTG | TGGATAAATAGCCAGCCCGA |
| circDENND1B | rat | AGTTTGTTTGTGTATTCTTAGATC | GACGAAGGTAAAGTGCTGTC |
| circEPHA5 | rat | GGGCCTACAGATCAGTAGGT | CCACATTCGCAGCAATCCAA |
| circMED13 | rat | AAGCTGTAGTTGTTTGAGTGAA | TGGGCTATTAGACTGCTGAG |
| circUBN2 | rat | GAAGAAATGCTTCTGTGCAAAA | TTAAATCCTGCAGCCGATCC |
| circEDIL3 | rat | ACCAGTCACAATGGTTACAGATA | ATTGCCACGGAAGACCATTT |
| circNUP88 | rat | GCATCCTGCAGCTGAAGATA | CTCACGCAGAGAGTAAATTCG |
