# Supplementary material for: Circular RNA METTL9 contributes to neuroinflammation following traumatic brain injury by complexing with astrocytic SND1
Source: J Neuroinflammation. 2023 Feb 17;20:39. doi: 10.1186/s12974-023-02716-x (PMC9936775; doi:10.1186/s12974-023-02716-x)
Supplement: Supplementary file 7 — Additional file 7: Table S7. RT-PCR and agarose gel electrophoresis of 7 circRNAs which were amplified by specific primers. [file 12974_2023_2716_MOESM7_ESM.docx]

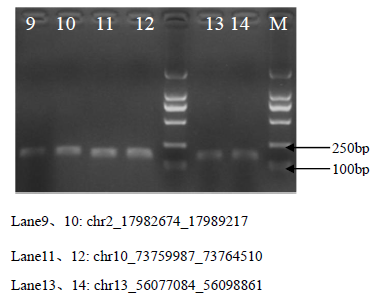

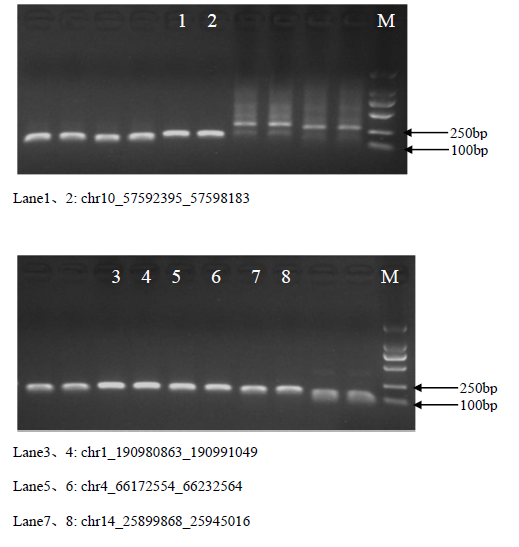
**Supplementary Table 7. RT-PCR and agarose gel electrophoresis of 7 circRNAs which were amplified by specific primers.**

chr10_57592395_57598183 is circNUP88

chr2_17982674_17989217 is circEDIL3

chr13_56096833_56173172 is circDENND1B

chr14_25899868_25945016 is circEPHA5

chr1_190980863_190991049 is circMETTL9

chr4_66172554_66232564 is circUBN2

chr10_73759987_73764510 is circMED13
