# Supplementary material for: Circular RNA METTL9 contributes to neuroinflammation following traumatic brain injury by complexing with astrocytic SND1
Source: J Neuroinflammation. 2023 Feb 17;20:39. doi: 10.1186/s12974-023-02716-x (PMC9936775; doi:10.1186/s12974-023-02716-x)
Supplement: Supplementary file 8 — Additional file 8: Table S8. MiRNAs that have the potential to bind to circMETTL9. [file 12974_2023_2716_MOESM8_ESM.docx]

**Supplementary Table 8. MiRNAs that have the potential to bind to circMETTL9.**

| circRNA | miRNA | mreFreq |
| --- | --- | --- |
| circMETTL9 | rno-miR-551b-5p | 2 |
|  | rno-miR-6315 | 2 |
|  | rno-miR-664-3p | 1 |
|  | rno-miR-294 | 1 |
